# Preoperative diagnosis of meningioma sinus invasion based on MRI radiomics and deep learning: a multicenter study

**DOI:** 10.1186/s40644-025-00845-5

**Published:** 2025-02-28

**Authors:** Yuan Gui, Wei Hu, Jialiang Ren, Fuqiang Tang, Limei Wang, Fang Zhang, Jing Zhang

**Affiliations:** 1 Department of Radiology, The Fifth Affiliated Hospital of Zunyi Medical University, zhufengdadao No.1439, Zhuhai, Doumen District China; 2 School of Medical Imaging, Zunyi Medical University, Zunyi, China; 3 Department of Pharmaceuticals Diagnosis, GE Healthcare, Beijing, China; 4 School of Nursing, Zunyi Medical University, Zunyi, China

**Keywords:** Meningioma, Sinus invasion, Radiomics, Deep learning

## Abstract

**Objective:**

Exploring the construction of a fusion model that combines radiomics and deep learning (DL) features is of great significance for the precise preoperative diagnosis of meningioma sinus invasion.

**Materials and methods:**

This study retrospectively collected data from 601 patients with meningioma confirmed by surgical pathology. For each patient, 3948 radiomics features, 12,288 VGG features, 6144 ResNet features, and 3072 DenseNet features were extracted from MRI images. Thus, univariate logistic regression, correlation analysis, and the Boruta algorithm were applied for further feature dimension reduction, selecting radiomics and DL features highly associated with meningioma sinus invasion. Finally, diagnosis models were constructed using the random forest (RF) algorithm. Additionally, the diagnostic performance of different models was evaluated using receiver operating characteristic (ROC) curves, and AUC values of different models were compared using the DeLong test.

**Results:**

Ultimately, 21 features highly associated with meningioma sinus invasion were selected, including 6 radiomics features, 2 VGG features, 7 ResNet features, and 6 DenseNet features. Based on these features, five models were constructed: the radiomics model, VGG model, ResNet model, DenseNet model, and DL-radiomics (DLR) fusion model. This fusion model demonstrated superior diagnostic performance, with AUC values of 0.818, 0.814, and 0.769 in the training set, internal validation set, and independent external validation set, respectively. Furthermore, the results of the DeLong test indicated that there were significant differences between the fusion model and both the radiomics model and the VGG model (*p* < 0.05).

**Conclusions:**

The fusion model combining radiomics and DL features exhibits superior diagnostic performance in preoperative diagnosis of meningioma sinus invasion. It is expected to become a powerful tool for clinical surgical plan selection and patient prognosis assessment.

**Supplementary Information:**

The online version contains supplementary material available at 10.1186/s40644-025-00845-5.

## Introduction

According to the latest data from the Central Brain Tumor Registry of the U.S., meningioma is a common primary central nervous system tumor, comprising approximately 39.7% of intracranial tumors [1]. Due to their specific growth locations, meningiomas often have a close relationship with adjacent venous sinuses and are prone to infiltrating or encasing major intracranial venous systems, including the superior sagittal and transverse sinuses, with an incidence rate of approximately 14.6%−16.5% [2]. If meningiomas invade the venous sinus wall or extend into the main sinus cavity, especially when adjacent to important large draining veins and collateral anastomotic veins formed by compensation, this increases the difficulty of complete surgical resection [3]. Additionally, for surgeries involving parasagittal meningiomas, preserving important collateral draining veins is crucial, as failure to do so can result in severe complications such as intraoperative bleeding and postoperative neurological dysfunction. However, incomplete tumor resection may lead to residual tumor tissue around the veins, causing a higher recurrence rate. Therefore, precise preoperative diagnosis of meningioma sinus invasion is essential for surgical planning, reducing intraoperative and postoperative complications, and ultimately improving the prognosis for patients with meningioma.

Currently, for patients with meningioma invading the sinus, careful preoperative planning of venous circulation patterns is crucial for surgical planning. Imaging plays a crucial role in preoperative identification of meningioma sinus invasion. Commonly used techniques include digital subtraction angiography (DSA), computed tomography venography (CTV), and magnetic resonance venography (MRV), and so on. However, each of these techniques has its own corresponding disadvantages. DSA is not only costly but also an invasive procedure; CTV requires the injection of contrast agents, which to some extent limits its clinical application; MRV has limitations in image clarity when displaying smaller veins. Given the limitations of these techniques, it is particularly important to seek a more precise, safe, and efficient new technology for preoperative assessment of meningioma sinus invasion.

According to the guidelines of the European Society for Neuro-Oncology, MRI is recommended as the primary diagnostic method for meningiomas [4]. Due to its high soft tissue resolution, MRI is often used as an important imaging examination method in clinical practice. Moreover, previous studies have used MRI combined with radiomics to diagnose brain invasion, bone invasion, and sinus invasion of meningiomas [5–7], showing high diagnostic performance. Radiomics is a technology that extracts high-throughput quantitative features from medical imaging images, which can reflect the biological characteristics and heterogeneity of tumors. Furthermore, DL, as a frontier field of machine learning, can directly and automatically learn features from a large amount of input information to complete target tasks such as classification, reducing the preprocessing steps of manually extracting data features. Recent studies have shown that radiomics or DL performs well in the prediction of meningioma grading, typing, differential diagnosis, and pathological molecular expression [8–12]. These studies also indicate that radiomics and DL based on MRI can serve as potential methods for diagnosing meningioma sinus invasion.

As of the present, there have been studies using radiomics methods to diagnose meningioma sinus invasion preoperatively [7, 13], but no research has yet employed a combined approach of radiomics and DL for diagnosing sinus invasion. Therefore, building on this foundation, this study integrates DL methods to mine deeper-level image features, achieve visualization of tumor heterogeneity, stratify preoperative surgical risk for meningioma patients, and ultimately realize individualized and precise diagnosis and treatment for meningioma patients, which has significant clinical implications.

## Materials and methods

### Case collection

**Fig. 1:**
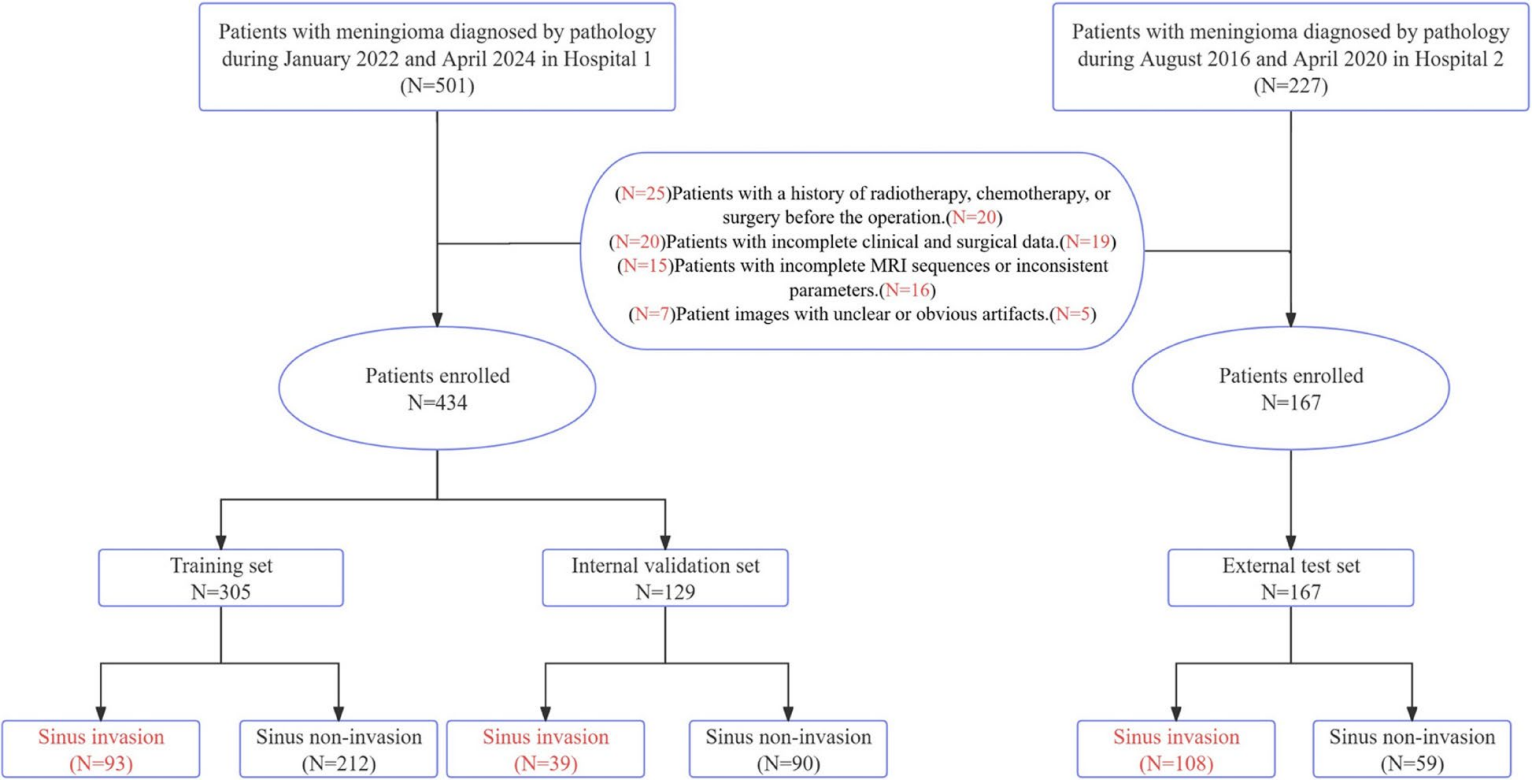
Inclusion and exclusion criteria

All cases in this case–control study were approved by the ethics review committees of Affiliated Hospital of Zunyi Medical University (Hospital 1) and Lanzhou University Second Hospital (Hospital 2). Given the retrospective nature of this study, informed consent from patients was waived. We retrospectively collected cases from both hospitals where patients had undergone surgical treatment and were pathologically confirmed to have meningioma, in accordance with preset inclusion and exclusion criteria. (Fig. 1) Patients from Hospital 1 were randomly assigned to a training set and an internal validation set in a 7:3 ratio. The training set included 305 patients, and the internal validation set included 129 patients. The 167 patients from Hospital 2 served as the independent external validation set. Using the surgical records of neurosurgeons during the operation as the criterion for diagnosing sinus invasion, neurosurgeons classified parasagittal meningioma into three types using the Simpson method [14]: Type I, tumor invading the venous sinus wall; Type II, tumor growing into the venous sinus with localized stenosis of the sinus cavity; Type III, tumor growing into the venous sinus with complete occlusion of the sinus cavity. Based on the histological characteristics of the venous sinus and surgical experience, we included Simpson Type I-III parasagittal meningioma in the sinus invasion group. Ultimately, the sinus invasion group included 240 patients, while the sinus non-invasion group included 361 patients. For specific inclusion and exclusion criteria, please refer to the supplementary document.

### MR image acquisition

All patients underwent plain and enhanced MRI scans one week before surgery, and MR images were obtained from the PACS system in DICOM format. The MR examinations were performed using 3.0-T scanners (Siemens Verio, Siemens Trio Tim) and 1.5-T scanners (Siemens Magnetom Aera) from the two hospitals. The MR images from each hospital included contrast-enhanced T1-weighted imaging (T1C), T2-weighted imaging (T2WI), and diffusion-weighted imaging (DWI) sequences. The detailed scanning parameters for each scanner are shown in Supplementary Table S1.

### MRI image segmentation

In this study, a manual segmentation method was used to precisely segment the lesions within the region of interest (ROI). Two radiologists, A and B, with 10 and 12 years of experience in MRI neuroimaging diagnosis, respectively, used ITK-Snap software (version 3.6.0; www.itksnap.org) to delineate the entire tumor as the volume of interest (VOI) across all sequences. The ROI focused on the main tumor area, excluding peritumoral edema, and was performed without knowledge of surgical and pathological records. A radiologist C, with 19 years of extensive experience in neuroimaging diagnosis, validated the segmentation results. For T2WI images, if the tumor boundaries were unclear, the morphology was irregular, or some DWI sequences did not adequately display the tumor lesion area, FLAIR and T1C sequences were used as auxiliary for separate delineation. After manual segmentation, due to the sensitivity of radiomics and DL features to the collected data, different MR scanning equipment and varying parameters of the same equipment could affect feature stability. To achieve image intensity normalization and discretization, z-score standardization was applied to preprocess T1C, T2WI, and DWI sequence images. N4ITK correction was used to correct bias field distortion. After bone removal, T2WI and T1C voxels were resampled to 1 × 1x1 mm^3^, DWI voxels were resampled to 1.5 × 1.5x1.5 mm^3^, and we divided the image grayscale into intervals of size 5 intensity units. To assess the reproducibility and robustness of the extracted features, 50 patients from the training set were randomly selected again after 3 months and re-delineated by radiologists A and B. Intra-class and inter-class correlation coefficients (ICCs) were used to evaluate the consistency between measurers, among measurers, and between different MR scanners.

### Radiomics and DL feature extraction

Each MRI sequence encodes different physical contrasts, and extracting features individually can leverage the unique biological or pathophysiological clues of each sequence. Furthermore, extracting features from each sequence one by one can better reduce the risk of overfitting and feature redundancy, and contribute to clearer biological interpretations. Therefore, we choose to extract radiomics and DL features from each MRI sequence separately and then combine them. The feature extraction algorithms were standardized in accordance with the Image Biomarker Standardisation Initiative [15]. 1) Radiomics feature extraction: PyRadiomics software was used to extract and quantify radiomics features from the VOIs delineated on T1C, T2WI, and DWI images [16]. A total of 3948 radiomics features were extracted from all VOIs, which mainly included three types of features: morphological features, histogram features, and texture features. There were 42 morphological features, 756 histogram features, and 3150 texture features. Among the 3150 texture features, there were 1008 Gray Level Co-occurrence Matrix (GLCM) features, 588 Gray Level Dependence Matrix (GLDM) features, 672 Gray Level Run Length Matrix (GLRLM) features, 672 Gray Level Size Zone Matrix (GLSZM) features, and 210 Neighbouring Gray Tone Difference Matrix (NGTDM) features.

**Fig. 2:**
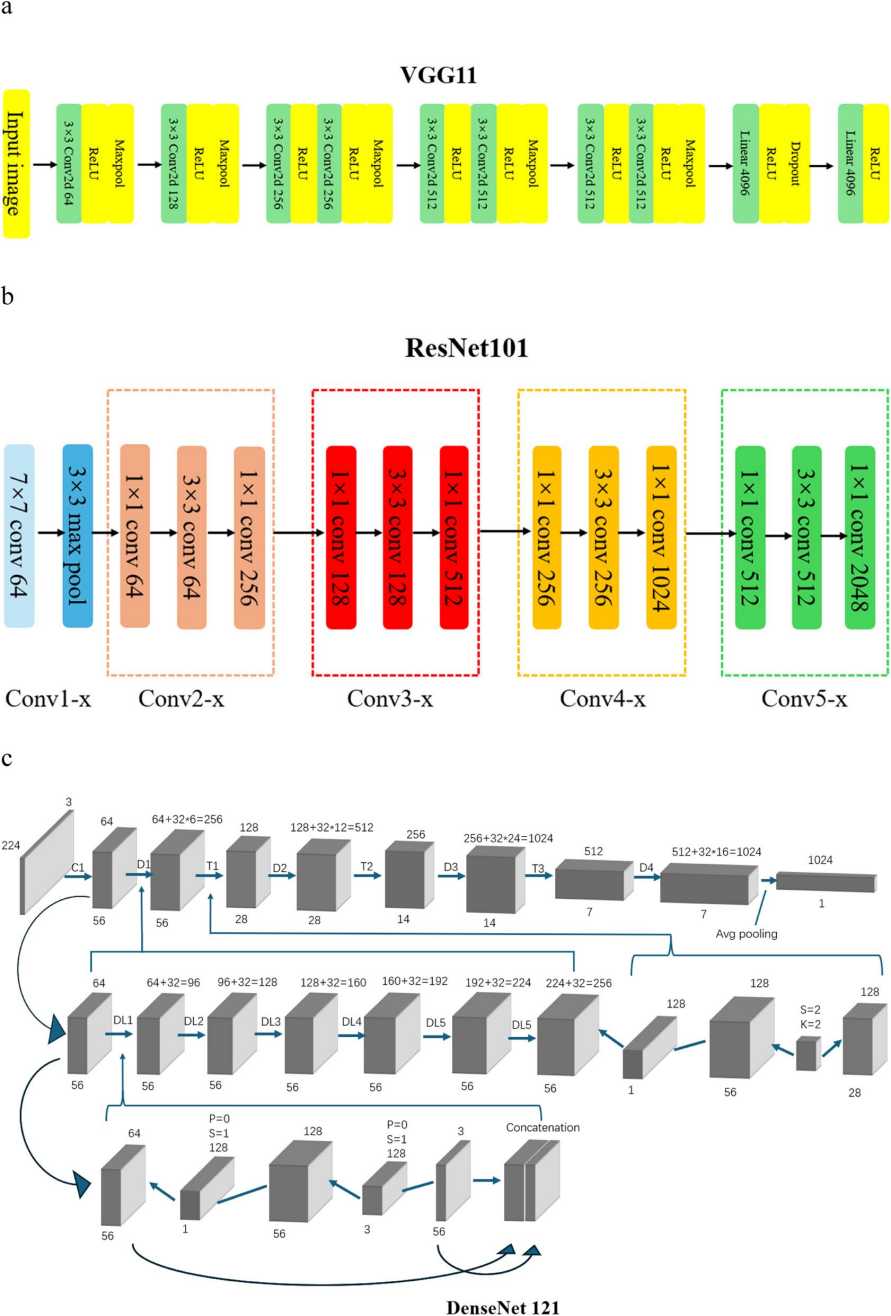
VGG11 (**a**), ResNet101 (**b**), and DenseNet121 (**c**) Network architecture figure

2) DL feature extraction: Apply VGG network, ResNet network, and DenseNet network for DL feature extraction. We extracted a representative two-dimensional image (224 × 224 pixels) from the VOIs of each tumor, specifically the image of the largest cross-sectional area of the tumor within the VOIs, and input it into the corresponding DL networks for feature extraction. Ultimately, 12,288 VGG11 features, 6144 ResNet101 features, and 3072 DenseNet121 features were extracted, respectively. For the specific DL network architecture, please refer to Fig. 2, and for the parameters, please refer to the supplementary file Table S2-4.

### Feature selection

Firstly, ICCs were used to evaluate the intra- and inter-observer agreement, with values greater than 0.75 indicating good agreement. For all data in the training set, univariate logistic regression was applied to the extensive omics features calculated from the tumor ROI to retain features with* p* < 0.05. Next, correlation analysis was used to eliminate features with a correlation coefficient greater than 0.8. Finally, the Boruta algorithm was employed for further dimension reduction to decrease the number of features, thereby screening out features highly correlated with sinus invasion in meningioma and achieving feature selection. The stable and reproducible key features ultimately selected served as radiomics and DL signatures.

### Model construction and evaluation

**Fig. 3:**
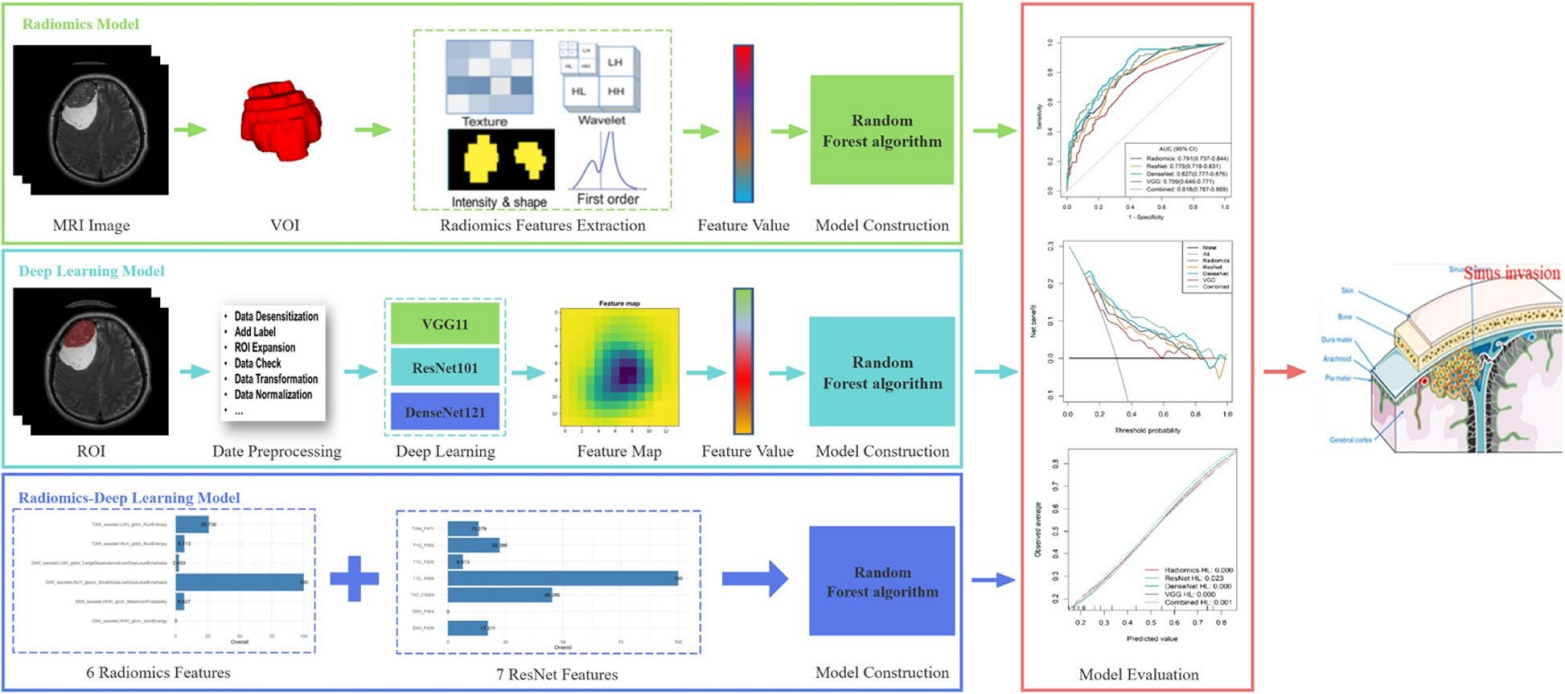
Technology roadmap

All selected features were utilized for model construction, with the training set used for model building and the validation set solely for model verification. To enhance the discriminatory power of radiomics and DL models, the selected T1C, T2WI, and DWI radiomics features were combined to construct radiomics signatures, and DL features were combined to construct DL signatures, comprehensively reflecting the factors affecting sinus invasion from different perspectives. Based on the constructed signatures, four single diagnosis models (radiomics model, VGG model, ResNet model, and DenseNet model) were built using the RF algorithm. ROC were plotted to evaluate the performance of different models in diagnosing sinus invasion in meningioma. Subsequently, the optimal DL model was selected and combined with the radiomics model constructed using radiomics signatures to build a DLR fusion model. Calibration curves were used to demonstrate the comparison between the diagnosis model and perfect fit, and the Hosmer–Lemeshow (H–L) test was employed to evaluate the diagnostic performance of the model. Additionally, decision curves were applied to quantify the net benefit at different threshold probabilities, assessing the clinical utility of the diagnosis model. A flowchart of the study is shown in Fig. 3.

### Statistical analysis

This study used Python (version 3.7.6), R software (version 4.1.1), and IBM SPSS 29.0 (IBM Corp) for all statistical analyses. The discrimination of the model was quantified by the AUC in the training set and validated in an independent external validation set. The AUC values, accuracy, sensitivity, and specificity of the different models in diagnosing sinus invasion in meningioma were compared. The diagnostic performance of different diagnosis models was compared using the DeLong test. Calibration curves were used to demonstrate the comparison between the diagnosis model and perfect fit, and the H–L test was used to assess whether there were significant differences between the diagnosis models and perfect fit. Decision curves were applied to quantify the net benefit at different threshold probabilities, assessing the clinical utility of the diagnosis model. ICCs were used to assess the consistency of features extracted by two radiologists and different MR scanners, respectively. *p* < 0.05 indicated statistical significance.

## Results

### Radiomics and DL feature selection

**Fig. 4:**
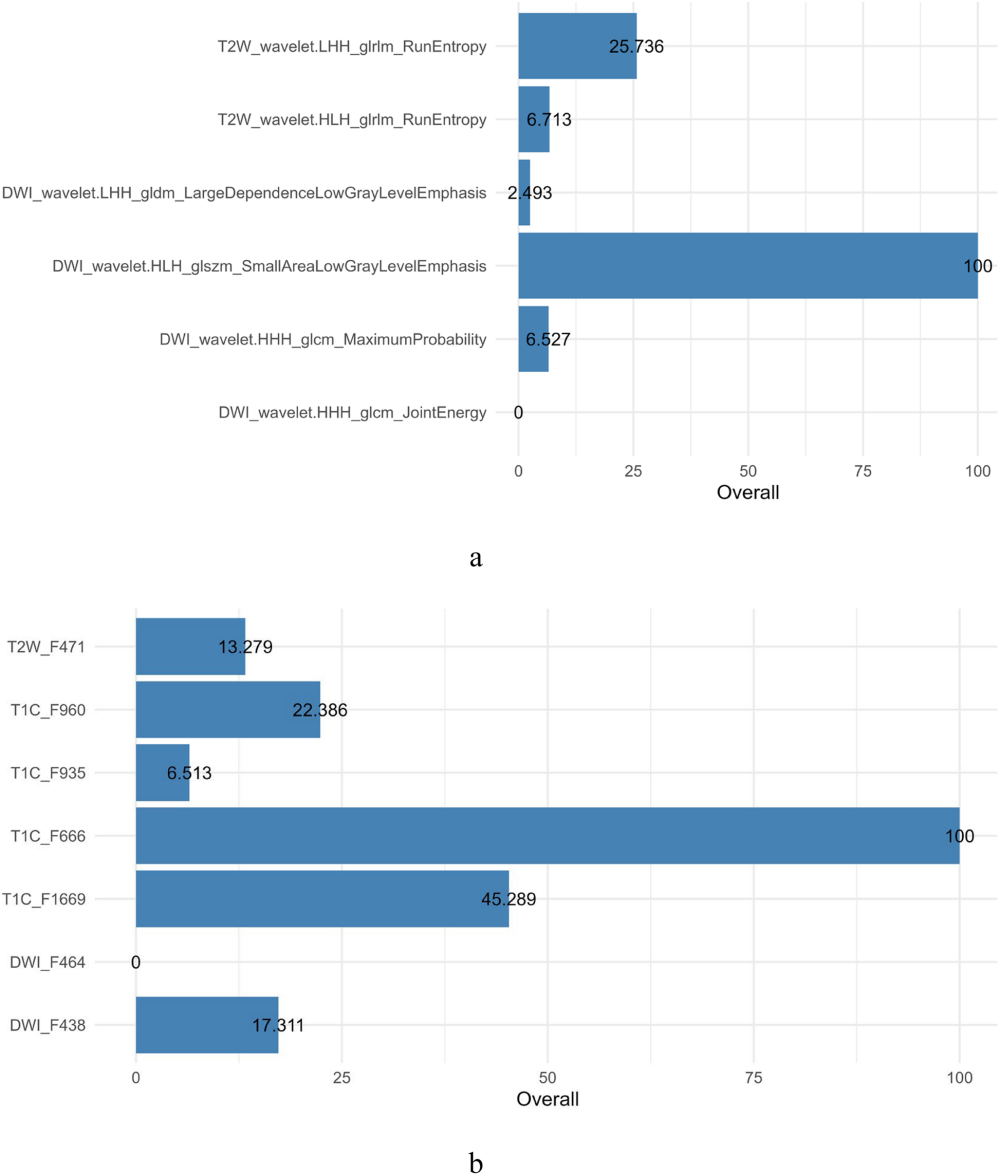
Radiomics and deep learning feature map. a: Radiomics features that were significantly associated with meningiomas sinus invasion. b: Deep learning features that were significantly associated with meningiomas sinus invasion

We assessed the reliability of all extracted radiomic features using ICCs. Across the entire set of 3948 features, the mean ± standard deviation (SD) of the intra-observer ICCs was 0.86 ± 0.07, whereas the mean ± SD of the inter-observer ICCs was 0.84 ± 0.08. After dimension reduction, a total of 6 radiomic features were retained. These selected features demonstrated higher reproducibility, with mean ± SD ICCs of 0.90 ± 0.05 for intra-observer measurements and 0.88 ± 0.06 for inter-observer measurements, indicating excellent agreement (all ICCs > 0.75). 1) Radiomics feature selection: Initially, 197 features were retained using univariate analysis; then, 15 features remained after correlation analysis; finally, six radiomics features (two T2WI features and four DWI features) highly correlated with sinus invasion were retained through Boruta analysis. The radiomics features are shown in Fig. 4a. 2) DL feature selection: VGG network, ResNet network, and DenseNet network were applied separately, and 12,288, 6144, and 3072 features were extracted, respectively. Initially, 81, 171, and 95 features were retained using univariate analysis, respectively; then, 3, 15, and 7 features remained after correlation analysis, respectively; finally, 2, 7, and 6 features highly correlated with sinus invasion remained after Boruta analysis, respectively. Construct a fusion model using seven features (one T2WI, four T1C, and two DWI features) from the DL model with the best performance. The DL features are shown in Fig. 4b.

### Diagnostic performance of different models

**Fig. 5:**
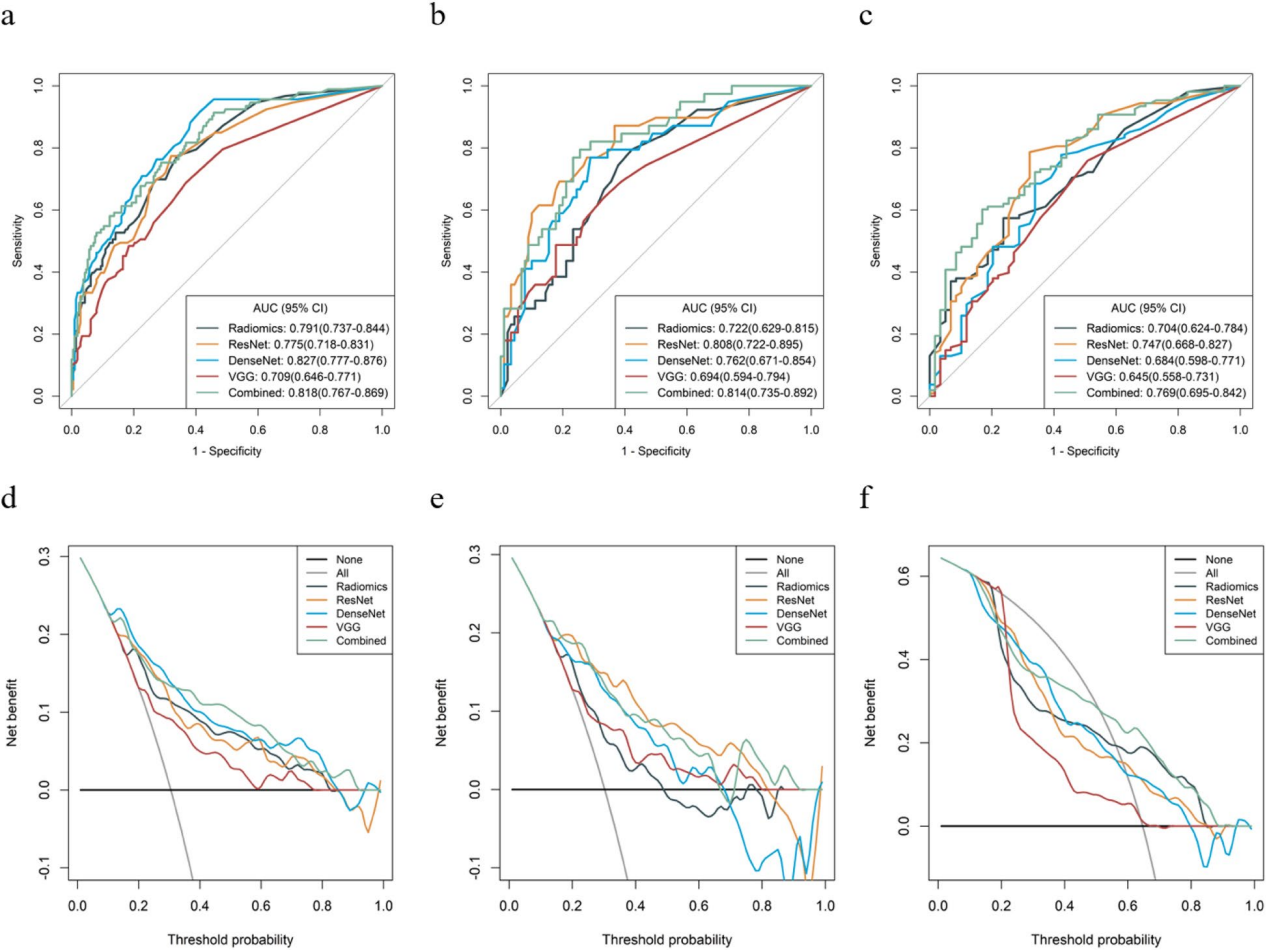
**a**-**c** ROC curves of the different models in the training set, internal validation set, and external validation set. **d**-**f** Decision curve analysis for the different model in the training set, internal validation set, and external validation set. The x-axis shows the threshold probability, and the y-axis measures the net benefit. The gray line represents all patients with sinus invasion, while the black line represents all patients without sinus invasion. The green line represents the combined model. The red, yellow, light blue, and dark blue lines represent VGG, ResNet, DenseNet, and Radiomics models, respectively

Due to the different principles of T1C, T2WI, and DWI sequences, their radiomics and DL features may reflect different information. By combining the selected radiomics features to construct radiomics signatures and combining the DL features to construct DL signatures, modal fusion is achieved, thereby reflecting the influencing factors of sinus invasion from different perspectives. Four single diagnosis models (radiomics model, VGG model, ResNet model, and DenseNet model) were constructed using the RF algorithm. Their discrimination ability was first evaluated in the training set and then validated in the internal validation set and independent external validation set. Among them, the model based on seven ResNet features showed good diagnostic performance, with AUC in the training set, internal validation set, and independent external validation set being 0.775 (0.718–0.831), 0.808 (0.722–0.895), 0.747 (0.668–0.827), respectively. Then, the best model was combined with the radiomics model constructed using six radiomics features to build a DLR fusion model, which further improved the diagnostic performance. The AUC in the training set, internal validation set, and independent external validation set were 0.818 (0.767–0.869), 0.814 (0.735–0.892), 0.769 (0.695–0.842), respectively. The AUC values, accuracy, sensitivity, specificity of different diagnosis models are shown in Table 1. The ROC curves of different diagnosis models are shown in Fig. 5a-c.

**Table 1:** Comparison of ROC curves for different models

	AUC	ACC	SN	SP	PPV	NPV
**Train set**						
Radiomics	0.791	0.695	0.774	0.660	0.500	0.870
ResNet	0.775	0.708	0.774	0.679	0.514	0.873
DenseNet	0.827	0.689	0.914	0.590	0.494	0.940
VGG	0.709	0.649	0.688	0.632	0.451	0.822
Combined	0.818	0.725	0.753	0.712	0.534	0.868
**Internal validation set**						
Radiomics	0.722	0.643	0.795	0.578	0.449	0.867
ResNet	0.808	0.690	0.795	0.644	0.492	0.879
DenseNet	0.762	0.574	0.872	0.444	0.405	0.889
VGG	0.694	0.636	0.692	0.611	0.435	0.821
Combined	0.814	0.744	0.821	0.711	0.552	0.901
**External validation set**						
Radiomics	0.704	0.635	0.722	0.475	0.716	0.483
ResNet	0.747	0.749	0.787	0.678	0.817	0.635
DenseNet	0.684	0.671	0.861	0.322	0.699	0.559
VGG	0.645	0.617	0.630	0.593	0.739	0.467
Combined	0.769	0.701	0.778	0.559	0.764	0.579

*ACC* balanced accuracy, *AUC* area under receiver operating characteristic curve, *SN* sensitivity, *SP* specificity, *PPV* positive predictive value, *NPV* negative predictive value. Combined, including the radiomics features and ResNet features

### Performance comparison of diagnosis models

The DeLong test was used to compare the discriminatory ability of AUC values between different models. When comparing the DLR fusion model with the radiomics model, the result showed z = −2.323, *p* < 0.05; when comparing it with the VGG model, z = −2.860, *p* < 0.05; when comparing it with the ResNet model, z = −0.649, *p* = 0.516; and when comparing it with the DenseNet model, z = −1.506, *p* = 0.132. The results indicated that there were significant differences between the fusion model and both the radiomics model and the VGG model (*p* < 0.05). A comprehensive comparison and analysis of AUC values for different models are shown in Table 2 and Table S5.

**Table 2:** Performance comparison between different models

Different models		Delong test	
		*p* value	z value
Combined	Radiomics	*p*<0.05	-2.323
	ResNet	0.516	-0.649
	DenseNet	0.132	-1.506
	VGG	*p*<0.05	-2.860

Combined, including the radiomics features and ResNet features. *p*<0.05 was considered to be significantly different

### Performance evaluation of diagnosis models

**Fig. 6:**
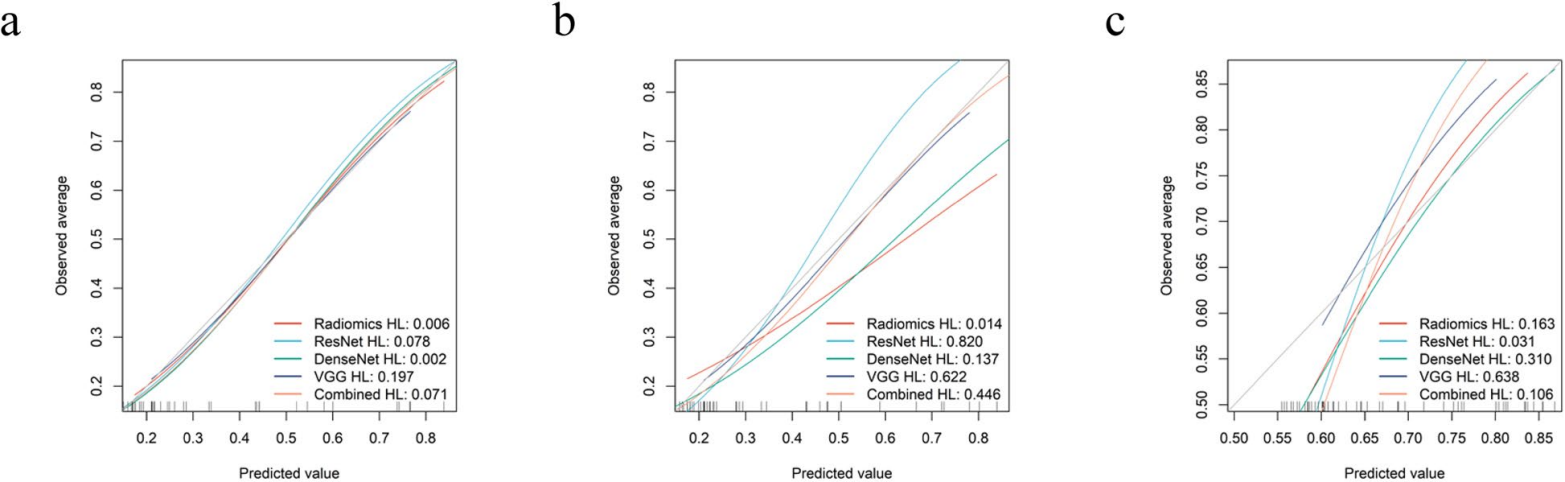
Calibration curves of the different models for the training set (**a**), internal validation set (**b**), and external validation set (**c**). The x-axis represents the probability of sinus invasion measured using different models, and the y-axis represents the actual rate of sinus invasion

The H–L test was used to evaluate the consistency between the actual sinus invasion of meningiomas and the diagnostic probability of sinus invasion by various models. As shown in Fig. 6, the results indicated that their diagnosis was consistent with the actual probability of sinus invasion. Additionally, the decision curve assessed the discriminatory ability of each model, and the results showed that they had high stability and clinical practicality. The decision curve analysis (Fig. 5d-e) provided the net benefit of diagnosing sinus invasion of meningiomas by each model, with a threshold probability greater than 20%.

## Discussion

Until now, this represents the first study to develop a radiomics and DL fusion model based on multicenter MRI data for preoperative diagnosis of meningioma sinus invasion. The performance of this fusion model was validated through calibration curves and decision curve analysis in both the internal and external validation sets. 6 radiomic features and 7 DL features were found to be highly correlated with sinus invasion and maintained stability across multiple centers. The results demonstrated that the fusion model combining radiomic and DL signatures exhibited the highest diagnostic performance ( AUC values of 0.818, 0.814, and 0.769 in the training set, internal validation set, and independent external validation set, respectively). It is capable of accurately identifying whether sinus invasion has occurred preoperatively, which greatly assists clinicians in formulating treatment plans.

In this study, we analyzed the correlation between radiomic features and sinus invasion, identifying two GLRLM features, one GLSZM feature, two GLDM features, and two GLCM features that were significantly associated with sinus invasion. Unlike previous studies [7, 13], we found a significant correlation between the GLSZM feature and sinus invasion. This feature quantifies the distribution of different gray-level zones within the image, thereby revealing the texture structure and heterogeneity of the image. Moreover, research has shown that GLRLM, GLCM, and GLDM features are highly correlated with brain and bone invasion [5, 6], respectively, which may suggest their relevance to aggressive meningiomas. Therefore, radiomic features can serve as novel biomarkers for preoperative diagnosis of meningioma sinus invasion.

With the rapid development of artificial intelligence, DL has achieved remarkable success in the field of medical image analysis [17–19]. Moreover, studies have shown that DL features can enhance the accuracy of radiomics models [20–22]. Through comparative analysis of four individual models, it was found that the ResNet network demonstrated the best performance in both the internal and external validation sets (AUC: 0.808, 0.747). Furthermore, calibration curve analysis revealed that, compared to the other three models, the ResNet model exhibited relatively better calibration, with the curve generally approaching the diagonal line. This may be attributed to the fact that, in contrast to radiomics, VGG, and DenseNet networks, the ResNet network addresses the vanishing gradient problem through residual connections, enabling the training of very deep network structures without performance degradation. Additionally, the presence of residual connections renders the ResNet network more stable during training, reducing the likelihood of gradient explosion issues. Lastly, it also serves as a form of regularization, aiding in the prevention of overfitting and enhancing the model's generalizability. In summary, the results substantiate that the ResNet network architecture may perform more optimally in diagnosing sinus invasion, thereby improving the model's training efficiency and diagnostic performance to a certain extent. This also provides a reference for the selection of DL network architectures in future research.

Previously, our studies have utilized radiomics based on MRI and a combination of radiomics with both MRI and DWI for preoperative diagnosis of meningioma sinus invasion, demonstrating promising performance [7, 13]. Building on previous research, we incorporated DL methods for preoperative diagnosis of meningioma sinus invasion, and this study also has unique advantages. One of the key methodological innovations of this study is the comprehensive evaluation of multiple DL architectures (VGG, ResNet, and DenseNet) to capture different levels of feature abstraction—VGG for basic visual patterns, ResNet for complex hierarchical features, and DenseNet for dense feature reuse. This multi-architecture approach, combined with radiomic features, provides a more comprehensive characterization of tumor phenotypes than any previous single-method studies. Additionally, while previous studies had smaller sample sizes, our study included 601 cases from two centers, enhancing the generalizability of the model. Moreover, a distinctive aspect of our study is the extensive validation through both internal and external validation sets, whereas most previous studies were limited to single-center validation. These methodological advantages, particularly our novel multi-architecture DL approach, along with superior performance metrics, indicate that our fusion approach represents a significant step forward in the field of preoperative meningioma assessment.

In addition, previous studies have demonstrated promising performance of models constructed using conventional MRI combined with DWI sequences in breast cancer, hepatocellular carcinoma, gliomas, and so on [23–25]. Therefore, in this study, we integrated T1C, T2WI, and DWI sequences to construct a model. Each sequence encodes distinct physical contrasts. The T1C sequence is commonly used to assess blood supply and the extent of tumor infiltration, and it can delineate visible tumor boundaries [26]. The T2WI sequence, due to its sensitivity to water-containing tissues, can be employed to detect the presence of edema [27]. DWI, as a functional MRI imaging technique, is sensitive to the micrometer distances detected by the random motion of water molecules in a short time between two gradient pulses. It can explore tissue structure, vascular distribution, and microstructure at the sub-voxel level, revealing more intrinsic information about the disease [28, 29]. Combining these sequences allows for a more comprehensive reflection of the tumor's biological information. We chose to extract radiomics and DL features separately from each MRI sequence to fully utilize the unique biological information of each sequence, and this approach minimizes the risk of overfitting. Moreover, clinicians and radiologists often rely on different MRI sequences to highlight specific tumor characteristics. This method facilitates clearer biological interpretation and fully leverages the advantages of multi-sequence imaging, thereby generating a more stable and interpretable diagnostic model for preoperative meningioma sinus invasion.

However, the training data for this model are limited to patients with meningiomas who underwent surgical intervention and do not include those treated conservatively. This is because pathological diagnosis is the gold standard for confirming meningiomas, and tumor specimens are typically obtained only through surgery. For patients with meningiomas who receive conservative treatment, we mostly rely on imaging methods for diagnosis, which may be subject to subjective influences. Including patients with atypical imaging features of meningiomas in the model training may adversely affect the model’s performance. Moreover, in this study, when applying the model to patients treated conservatively, clinicians should integrate it with traditional imaging methods for a comprehensive assessment. In future studies, we will prospectively include patients with typical imaging features of meningiomas who are treated conservatively to further validate the model’s applicability.

This study also has some limitations. Firstly, since it is a retrospective study and we only included patients with meningiomas that were confirmed by surgical pathology, this may lead to selection bias. Secondly, in this study, we relied on experienced radiologists to manually delineate the regions of interest. While manual delineation has a high accuracy rate, this process requires a significant amount of effort and time from radiologists. Lastly, image scanning and reconstruction protocols often vary greatly between different equipment manufacturers and different models of imaging equipment from the same manufacturer, and there is currently a lack of unified acquisition standards.

## Conclusions

The fusion model constructed by combining radiomics and DL signatures has shown high effectiveness and clinical practicality in preoperative diagnosis of sinus invasion in meningiomas. This finding provides clinicians with a more precise and noninvasive preoperative assessment tool, which is helpful for optimizing surgical plans, reducing surgical risks, and improving patient outcomes. In the future, with the continuous advancement of technology, the application prospects of this model will be even broader.

## Authors' contributions

G.Y. was responsible for data collection and manuscript writing, H.W. was responsible for data collection and processing, R.J.L. was responsible for data analysis, T.F.Q., W.L.M., and Z.F. were responsible for the creation of figures, and Z.J. was responsible for manuscript review, project management, and funding acquisition. All authors have reviewed the manuscript.

## Supplementary Information

Supplementary Material 1.

## Data Availability

No datasets were generated or analysed during the current study.

## Abbreviations

DL: Deep learning
T2WI: T2-weighted imaging
T1C: Contrast-enhanced T1-weighted imaging
DWI: Diffusion-weighted imaging
RF: Random forest
ROC: Receiver operating characteristic
H–L: Hosmer–Lemeshow
AUC: Area under the curve
DLR: DL-radiomics
DSA: Digital subtraction angiography
CTV: Computed tomography venography
MRV: Magnetic resonance venography
ROI: Region of interest
VOI: Volume of interest
ICCs: Intra- and inter-class correlation coefficients
SD: Standard deviation
GLCM: Gray Level Co-occurrence Matrix
GLDM: Gray Level Dependence Matrix
GLRLM: Gray Level Run Length Matrix
GLSZM: Gray Level Size Zone Matrix
NGTDM: Neighbouring Gray Tone Difference Matrix
