# Supplementary material for: Preoperative diagnosis of meningioma sinus invasion based on MRI radiomics and deep learning: a multicenter study
Source: Cancer Imaging. 2025 Feb 28;25:20. doi: 10.1186/s40644-025-00845-5 (PMC11869444; doi:10.1186/s40644-025-00845-5)
Supplement: Supplementary file 1 — Supplementary Material 1. [file 40644_2025_845_MOESM1_ESM.docx]

**Supplementary material**

**I. Supplementary tables**

**II.** **Supplementary methods**

**Supplementary tables**

**Table.S1: The detailed parameters for each scanner.**

| Hospital | Scanner | Sequence | TR(ms) | TE(ms) | Slice Thickness(mm) | Slice Gap(mm) | FOV(cm) | Matrix |
| --- | --- | --- | --- | --- | --- | --- | --- | --- |
| Hospital1 | Siemens Verio | T1C | 250 | 2.48 | 5 | 1 | 22X22 | 256X256 |
|  |  | T2WI | 4000 | 96 | 5 | 1 | 22X22 | 256X256 |
|  |  | DWI | 4750 | 95 | 5 | 1 | 22X22 | 230X230 |
|  | Siemens Trio Tim | T1C | 245 | 2.50 | 5 | 1 | 22X22 | 256X256 |
|  |  | T2WI | 4050 | 100 | 5 | 1 | 22X22 | 256X256 |
|  |  | DWI | 4700 | 100 | 5 | 1 | 22X22 | 230X230 |
| Hospital2 | Siemens Magnetom Aera | T1C | 250 | 2.50 | 5 | 1 | 22X22 | 256X256 |
|  |  | T2WI | 4000 | 95 | 5 | 1 | 22X22 | 256X256 |
|  |  | DWI | 4800 | 95 | 5 | 1 | 22X22 | 230X230 |

Abbreviations: T1C, contrast-enhanced T1-weighted imaging; T2WI, T2-weight imaging; DWI, diffuse weighted imaging; TR, repetition time; TE, echo time.

**Table S2: VGG11 network architecture parameters.**

| Layer type | Layers | Number of input channels | Number of output channels | Size of the convolutional kernel | Stride | Padding | Size of the pooling kernel | Pooling stride | Activation function |
| --- | --- | --- | --- | --- | --- | --- | --- | --- | --- |
| Convolutional layer | 1 | 3 | 64 | 3×3 | 1 | 1 | - | - | ReLU |
| Max pooling layer | 1 | - | - | - | - | - | 2×2 | 2 | - |
| Convolutional layer | 1 | 64 | 128 | 3×3 | 1 | 1 | - | - | ReLU |
| Max pooling layer | 1 | - | - | - | - | - | 2×2 | 2 | - |
| Convolutional layer | 2 | 128 | 256 | 3×3 | 1 | 1 | - | - | ReLU |
| Max pooling layer | 1 | - | - | - | - | - | 2×2 | 2 | - |
| Convolutional layer | 2 | 256 | 512 | 3×3 | 1 | 1 | - | - | ReLU |
| Max pooling layer | 1 | - | - | - | - | - | 2×2 | 2 | - |
| Convolutional layer | 2 | 512 | 512 | 3×3 | 1 | 1 | - | - | ReLU |
| Max pooling layer | 1 | - | - | - | - | - | 2×2 | 2 | - |
| Fully connected layer | 1 | 4096 | 4096 | - | - | - | - | - | ReLU |

**Table S3: ResNet 101 network architecture parameters.**

| Layer type | Layers | Number of input channels | Number of output channels | Size of the convolutional kernel | Stride | Padding | Size of the pooling kernel | Pooling stride | Activation function |
| --- | --- | --- | --- | --- | --- | --- | --- | --- | --- |
| Convolutional layer | 1 | 3 | 64 | 7×7 | 2 | 3 | - | - | ReLU |
| Max pooling layer | 1 | - | - | - | - | - | 3×3 | 2 | - |
| Residual block group 1 | 3 | 64 | 256 | 1×1,3×3,1×1 | 1 | 1 | - | - | ReLU |
| Residual block group 2 | 4 | 256 | 512 | 1×1,3×3,1×1 | 2 | 1 | - | - | ReLU |
| Residual block group 3 | 23 | 512 | 1024 | 1×1,3×3,1×1 | 2 | 1 | - | - | ReLU |
| Residual block group 4 | 3 | 1024 | 2048 | 1×1,3×3,1×1 | 2 | 1 | - | - | ReLU |
| Global average pooling | 1 | - | - | - | - | 1 | 7×7 | - | - |

**Table S4: DenseNet 121 network architecture parameters.**

| Layer type | Layers | Number of input channels | Number of output channels | Size of the convolutional kernel | Stride | Padding | Size of the pooling kernel | Pooling stride | Activation function |
| --- | --- | --- | --- | --- | --- | --- | --- | --- | --- |
| Convolutional layer | 1 | 3 | 64 | 7×7 | 2 | 3 | - | - | ReLU |
| Max pooling layer | 1 | - | - | - | - | - | 3×3 | 2 | - |
| Dense block 1 | 6 | 64 | 256 | 1×1,3×3 | 1 | 1 | - | - | ReLU |
| Transition layer 1 | 1 | 256 | 128 | 1×1 | 1 | 0 | 2×2 | 2 | - |
| Dense block 2 | 12 | 128 | 512 | 1×1,3×3 | 1 | 1 | - | - | ReLU |
| Transition layer 2 | 1 | 512 | 256 | 1×1 | 1 | 0 | 2×2 | 2 | - |
| Dense block 3 | 24 | 256 | 1024 | 1×1,3×3 | 1 | 1 | - | - | ReLU |
| Transition layer 3 | 1 | 1024 | 512 | 1×1 | 1 | 0 | 2×2 | 2 | - |
| Dense block 4 | 16 | 512 | 1024 | 1×1,3×3 | 1 | 1 | - | - | ReLU |
| Global average pooling | 1 | - | - | - | - | - | 7×7 | - | - |

**Table S5: Performance comparison between different models.**

| Different models | | Delong test | |
| --- | --- | --- | --- |
|  |  | p value | z value |
| Radiomics | ResNet | 0.414 | -0.817 |
|  | DenseNet | 0.729 | 0.347 |
|  | VGG | 0.254 | 1.142 |
| ResNet | DenseNet | 0.275 | 1.091 |
|  | VGG | *p<*0.05 | 2.347 |
| DenseNet | VGG | 0.468 | 0.725 |

**Supplementary methods**

**Inclusion/exclusion criteria**

1) Inclusion criteria: a) Patients with pathologically confirmed WHO grade 1-3 meningioma according to the latest 2021 WHO Classification of Tumors of the Central Nervous System; b) All patients underwent MRI multi-sequence (T1C, T2WI, and DWI) scans one week before surgery, with clear and complete images; c) All patients underwent surgical resection with complete surgical assessment records (presence or absence of sinus invasion); d) All patients had complete pathological results (grading, typing, and immunohistochemistry). 2) Exclusion criteria: a) Patients with preoperative history of radiotherapy, chemotherapy, or surgery; b) Patients with incomplete clinical and surgical data; c) Incomplete MRI sequences or inconsistent parameters; d) Images with poor clarity or significant artifacts.

The main steps of Boruta analysis are as follows:

1. **Generation of Shadow Features**: For each feature in the original dataset, a shadow feature is created by randomly permuting the values.
2. **Training of Random Forest**: A random forest model is trained on the dataset that includes both the original and shadow features.
3. **Calculation of Importance**: The importance score for each feature (including shadow features) is calculated.
4. **Comparison of Importance**: The importance scores of the original features are compared with those of the shadow features to determine which features have significantly higher importance than the shadow features.
5. **Iterative Process**: The above steps are repeated multiple times until the importance scores of all features stabilize or the preset number of iterations is reached.
6. **Determination of Important Features**: Finally, it is determined which features are important, features with *p* < 0.05 are retained.
